# Senescence in head and neck squamous cell carcinoma: relationship between senescence-associated secretory phenotype (SASP) mRNA expression level and clinicopathological features

**DOI:** 10.1007/s12094-023-03364-6

**Published:** 2024-01-04

**Authors:** Kamila Ostrowska, Patryk Niewinski, Igor Piotrowski, Julia Ostapowicz, Sabina Koczot, Wiktoria Maria Suchorska, Paweł Golusiński, Michal Mateusz Masternak, Wojciech Golusiński

**Affiliations:** 1 Department of Head and Neck Surgery, Poznan University of Medical Sciences, 61-866 Poznan, Poland; 2 Radiobiology Laboratory, The Greater Poland Cancer Centre, 61-866 Poznan, Poland; 3 College of Medicine, Burnett School of Biomedical Sciences, University of Central Florida, Orlando, FL 32827 USA; 4 Department of Electroradiology, Poznan University of Medical Sciences, 61-866 Poznan, Poland; 5 Department of Otolaryngology and Maxillofacial Surgery, University of Zielona Góra, 65-417 Zielona Góra, Poland

**Keywords:** Head and neck squamous cell carcinoma, Cellular senescence, Senescence-associated secretory phenotype, Biomarkers

## Abstract

**Background:**

Cellular senescence is a state characterized by cell-cycle arrest and apoptotic resistance. Senescence in cancer may be induced by oncogenes or therapy. While cellular senescence might play an important role in protection against cancer development, elevated and uncontrolled senescent cells accumulation may promote carcinogenesis by secreting a collection of pro-inflammatory factors, collectively termed the senescence-associated secretory phenotype (SASP).

**Material and methods:**

We determined the gene expression at mRNA level of selected cellular senescence markers (p16 and LMNB1) and SASP factors (IL-6, IL-1b, CXCL-1 and TNF-α) in 72 cancerous tissues and 64 normal tissues obtained from patients with head and neck squamous cell carcinoma (HNSCC) and correlated this data with patients’ clinical follow-up.

**Results:**

Our results indicate higher levels of selected SASP factors in cancerous compared to normal tissues. We presented the relationship between SASP factors expression at the transcript level and the progression of the disease. Moreover, we proposed CXCL1 as a candidate biomarker differentiating normal tissues from cancerous ones and IL1b expression as a molecular factor related to increased TNM stage.

**Conclusion:**

Our primary study indicates that SASP expression may be associated with some clinicopathological features. However, a more detailed study is needed to present specific role of senescence-related mechanism and SASPs especially in tumor therapy response and in relation to the patient’s immune system condition.

**Supplementary Information:**

The online version contains supplementary material available at 10.1007/s12094-023-03364-6.

## Introduction

Head and neck squamous cell carcinomas (HNSCCs) develop from the mucosal epithelium in the oral cavity, pharynx and larynx and are the 7th most common malignancies worldwide. HNSCC accounts for more than 660,000 new cases and 325,000 deaths annually [1]. HNSCC is characterized by various genetic and epigenetic mutations and the presence of different types of cells. In response to various intrinsic and extrinsic stimuli (especially radiotherapy or definitive chemoradiotherapy) cancer cells may enter a state of permanent growth arrest known as cellular senescence [2, 3]. The impact of cellular senescence on tumorigenesis is dictated by the cell or tissue of origin and may be cancer specific [4]. The presence of senescent cells may be protective for tissue by inhibiting cancer cells proliferation and preventing the propagation of deleterious genetic mutations [5]. Moreover, senescent cells are metabolically active and can produce and release substances that affect the tumor microenvironment (TME), mainly through the production of senescence-associated secretory phenotype (SASP) or Senescence-Messaging Secretome (SMS) [2, 6]. The SASP factors include cytokines, chemokines, growth factors, and proteases. SASP factors are likely responsible for the negative aspects of cellular senescence in cancer through promote tumor development and relapse by creating an immunosuppressive environment [2, 6–9]. In this study we focused on expression of selected SASP factors such as IL6 (Interleukin 6), IL1b (Interleukin 1b), CXCL1 (C-X-C motif chemokine ligand 1), TNF-α (tumor necrosis factor α). In tumor biology, interleukins as IL-1b, IL-8, IL-6 promote cell proliferation and epithelial-mesenchymal transition (EMT), but IL-6 also influences cell cycle arrest and immune clearance of damaged cells, as well as CCL2 and CXCL1. CXCL1 and CXCL12 promote tumor cell migration, CXCL5 increases angiogenesis while TNF-α affects immune-mediated clearance [4, 5, 8]. As the induction of senescence-related process and production of SASP factors are related to resistance to cancer therapy, specifically to HNSCC radiotherapy, our investigation is in increasing interest for clinical management of this cancer. Moreover, there is a gap of knowledge concerning differences in senescence markers and SASPs expression in HNSCC patient’s tumor versus normal tissue in relation to clinicopathological features. Thus, our study contributes to a better understanding of the importance of the senescence-related factors in groups of HNSCC patients with different stages of tumor development that may be particularly important in the individual treatment approach. Further, tumor marker research is critical for the prognosis of HNSCC and holds the prospect of improving treatment outcomes through targeted therapy.

## Materials and methods

### Clinical data

The study cohort consist of 72 primary tumors, 64 normal epithelial specimens and 40 serum samples obtained from head and neck squamous cell carcinoma patients who underwent surgical tumor resection in the Department of Head and Neck Surgery, Poznan University of Medical Sciences, The Greater Poland Cancer Centre. Each patient’s clinicopathological characteristics are summarized in (Table 1). The exclusion criteria for this study involved second primary tumor, distant metastasis, previous radio- and/or chemotherapy and HPV infection. The procedures were approved by the Local Ethical Committee of Poznan University of Medical Sciences (Consent No. 521/21). Informed consent was obtained from each patient before tissue collection.

**Table 1:** Clinicopathological characteristic of each patient participated in the study

No	Age	Sex	Location	T	N	M	Grade	Tissue type obtained
1	64	M	Larynx	3	1	0	3	T, N
2	62	M	Larynx	4	2	0	2	T, N
3	64	M	Larynx	4	0	0	2	T, N
4	54	M	Oral cavity	2	0	0	2	T, N
5	63	F	Larynx	3	0	0	1	T, N
6	67	M	Oral cavity	3	2	0	2	T, N
7	57	M	Oral cavity	1	1	0	1	T, N
8	65	M	Larynx	4	1	0	3	T, N
9	54	M	Oral cavity	1	0	0	2	T, N
10	65	M	Larynx	4	2	0	3	T, N
11	61	M	Larynx	4	2	0	2	T, N
12	42	F	Larynx	4	2	0	2	T, N
13	40	F	Oral cavity	2	2	0	2	T
14	54	M	Oral cavity	2	2	0	2	T
15	70	M	Larynx	3	1	0	3	T, N
16	75	F	Oral cavity	2	1	0	2	T, N
17	63	M	Larynx	4	2	0	3	T, N
18	49	M	Oral cavity	2	0	0	2	T, N
19	62	M	Larynx	3	0	0	2	T, N
20	43	M	Larynx	3	2	0	2	T, N
21	45	M	Oral cavity	4	1	0	2	T
22	73	M	Larynx	4	1	0	2	T, N
23	61	M	Oral cavity	4	2	0	3	T
24	64	M	Oral cavity	3	1	0	2	T
25	62	M	Oral cavity	3	0	0	1	T, N
26	52	F	Oral cavity	2	2	0	2	T, N
27	62	M	Larynx	4	0	0	2	T, N
28	76	M	Larynx	4	2	0	2	T, N
29	47	M	Larynx	4	0	0	2	T, N
30	65	M	Larynx	1	0	0	2	T
31	69	M	Larynx	3	0	0	2	T, N
32	67	M	Larynx	3	0	0	2	N
33	69	M	Larynx	4	0	0	2	T, N
34	65	M	Larynx	4	2	0	2	T, N
35	61	F	Larynx	3	1	0	2	T, N
36	70	M	Oral cavity	3	1	0	1	T, N, S
37	45	M	Oral cavity	4	1	0	2	T, N, S
38	64	M	Oral cavity	3	2	0	2	T, N, S
39	62	M	Oral cavity	4	3	0	2	T, N, S
40	53	F	Oral cavity	3	3	0	3	T, N, S
41	66	M	Oral cavity	3	0	0	1	T, N, S
42	58	F	Larynx	3	2	0	3	T, N, S
43	71	M	Oral cavity	3	1	0	2	T, N
44	72	M	Larynx	4	3	0	1	T, N, S
45	63	M	Oral cavity	2	0	0	1	T, S
46	67	F	Oral cavity	1	1	0	2	T, S
47	63	M	Oral cavity	4	2	0	2	T, N, S
48	73	M	Oral cavity	3	0	0	1	T, N, S
49	42	F	Oral cavity	1	0	0	1	T, N, S
50	64	M	Oral cavity	3	1	0	2	T, S
51	50	M	Oral cavity	4	1	0	2	T, N, S
52	52	M	Larynx	3	1	0	2	T, N, S
53	64	M	Oral cavity	2	3	0	3	T, N, S
54	81	M	Larynx	4	0	0	3	T, N, S
55	56	M	Oral cavity	4	2	0	3	T, N
56	70	F	Oral cavity	4	0	0	2	T, N, S
57	65	F	Larynx	4	2	0	2	T, N, S
58	57	M	Larynx	4	0	0	2	T, N, S
59	71	M	Larynx	3	0	0	2	T, N, S
60	81	M	Larynx	4	3	0	2	T
61	59	M	Larynx	4	1	0	1	T, N, S
62	60	M	Larynx	3	0	0	1	T, N, S
63	59	M	Oral cavity	2	0	0	2	T, N, S
64	65	F	Oral cavity	3	2	0	2	T, N, S
65	51	M	Oral cavity	4	2	0	2	N, S
66	63	M	Oral cavity	3	0	0	1	T, N, S
67	69	M	Larynx	4	2	0	2	T, N, S
68	64	M	Larynx	4	1	0	2	T, N, S
69	57	F	Oral cavity	4	1	0	2	T, N, S
70	85	M	Larynx	4	2	0	3	T, N, S
71	65	M	Oral cavity	3	1	0	2	T, N, S
72	56	M	Oral cavity	1	0	0	2	T, N
73	63	M	Oral cavity	2	0	0	3	T, N, S
74	68	M	Oral cavity	2	3	0	2	T, N
75	67	M	Larynx	4	0	0	1	S
76	73	M	Larynx	4	1	0	2	S
77	70	M	Larynx	3	0	0	2	S
78	67	M	Larynx	4	0	0	3	S
79	62	M	Larynx	4	2	0	2	S
80	76	F	Larynx	4	0	0	2	S

*F* female, *M* male, *T* tumor tissue, *N* normal tissue, *S* serum

### RNA isolation, reverse transcription, and real-time quantitative polymerase chain reaction (RT-qPCR) analysis

The RNA purification kit (RNeasy Mini Kit, Qiagen, Hilden, Germany) was used to extract total RNA from tissue specimens. The cDNA was synthesized with RevertAid First Strand cDNA Synthesis Kit (ThermoFisher, Waltham, MA, USA) using 500 ng of total RNA, oligo dT primers, and random hexamer primers. The real-time quantitative polymerase chain reaction for *IL6, IL1b, CXCL1, TNF-α, LMNB1* and *P16* genes expression analysis was conducted with a PowerTrack SYBR Green Master Mix (ThermoFisher, Waltham, MA, USA) using the CFX96 Real-Time System (Bio-Rad, Hercules, CA, USA). The reaction conditions for all amplicons were as follows: initially 9 °C for 15 min, followed by 40 cycles at 95 °C for 10 s, 60–62 °C (depending on the primers used) for 10 s, and 72 °C for 10 s. The gene expression was normalized to the *18S* rRNA housekeeping gene, and relative expression levels were determined by the Pfaffl method. The primers sequence used in this study are listed in (Table S1).

### Enzyme-linked immunosorbent assay (ELISA)

To quantify CXCL1 protein in 40 serum samples, a specific ELISAs Kit (GRO alpha (CXCL1) Human ELISA Kit, ThermoFisher, Waltham, MA, USA) was used according to the manufacturer’s protocol. In brief, the microwell strips were washed with PBS with 1% Tween 20 and then serum samples and standards were added to each well. Afterwards, the sample were incubated with Biotin-Conjugate, Streptavidin-HRP, and substrate solution (tetramethyl-benzidine). The enzymatic reaction was terminated by addition of 1 M phosphoric acid, and the absorbance was measured at 450 nm. The standard curve was used to determine the concentration of CXCL1 values in the serum samples.

### Statistical analysis

First the outliers were identified and excluded from further analyses using the ROUT method (with Q = 1%). The normality of the observed patient data distribution was assessed using the Shapiro–Wilk test. The median expression values of cancerous and normal epithelial tissues were compared using the unpaired *t*-test (if data passed the normality test) or U Mann–Whitney test (if data did not pass the normality test). For the comparison of multiple groups, we used the one-way ANOVA with Tukey’s correction (if data passed the normality test) or Kruskal–Wallis’ test with Dunn’s correction (if data did not pass the normality test). To evaluate the accuracy of gene expression to distinguish cancerous from normal tissue, we produced the receiver operating characteristic (ROC) curves and calculated the area under the curve (AUC), sensitivity, and specificity for each ROC curve. For the above tests, the statistical analysis was performed using the GraphPad Prism v.8.1.1 software (GraphPad Software, Inc., La Jolla, CA, USA). The patients’ survival was analyzed by the Kaplan–Meier method. The optimal cutoff points of expression differentiating patients based on survival were determined using the Cutoff Finder application [9]. p < 0.05 was considered statistically significant.

## Results

### Quantitative analysis of SASP factors and senescence marker genes transcript levels in HNSCC patients

**Fig. 1:**
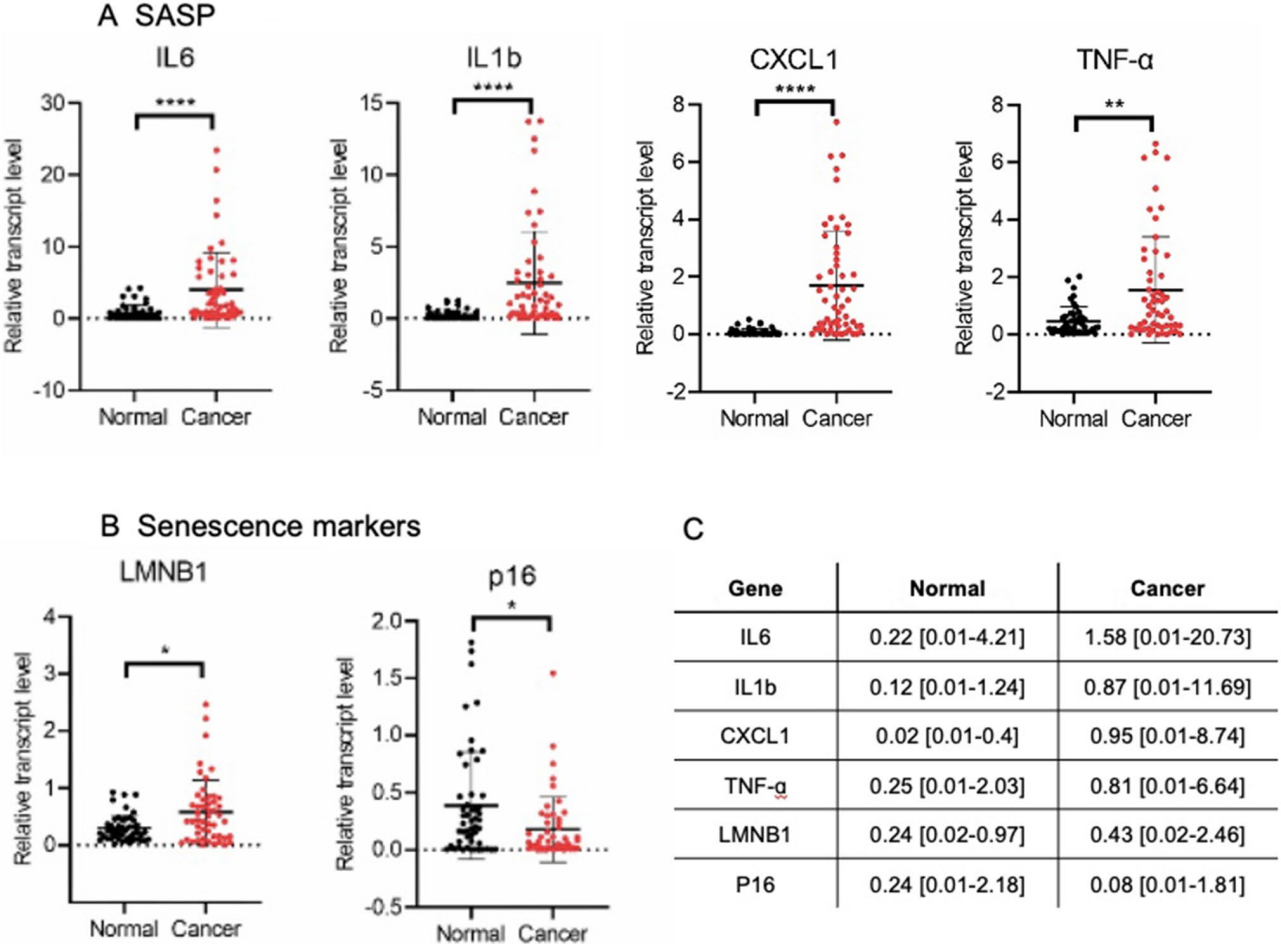
HNSCC cancer tissue exhibits higher mRNA expression of senescence-associated secretory phenotype (SASP) genes and altered expression of senescence marker genes compared to normal tissue. The transcript level of SASPs: *IL6*, *IL1b*, *CXCL1*, *TNF-α* (**A**) and senescence markers genes: *LMNB1*, *p16* (**B**) was measured in cancerous and normal tissues from HNSCC patients. **C** A median [with range] values of mRNA expression level for individual genes. Comparison of the two groups was performed by Mann–Whitney test, *p ≤ 0.05; **p < 0.01; ****p < 0.0001

72 cancerous and 64 normal tissues from patients with HNSCC were used to quantify the transcript level of selected SASP factors and senescence genes. (Table 2) summarize the clinicopathological features of analyzed patients. We found significantly higher mRNA levels in cancerous tissues compared to normal tissues of SASPs including *IL6* (p < 0.0001), *IL1b* (p < 0.0001), *CXCL1* (p < 0.0001), *TNF-α* (p = 0.0015). The analysis of senescence markers indicated significant upregulation of *LMNB1* (p = 0.0198) and *p16* (p = 0.0116) comparing cancerous and normal tissues (Fig. 1A, B).

**Table 2:** Characteristics of the study cohort

Characteristic	Total number [n (%)]
Age at the time of surgery (years)	
Mean	61.82
Median	63
Range	40–85
Gender	
Male	60 (81.08)
Female	14 (18.92)
Tumor stage (TNM classification)	
T1	7 (9.46)
T2	11 (14.86)
T3	25 (33.78)
T4	31 (41.89)
N0	26 (35.13)
N1	20 (27.03)
N2	22 (29.73)
N3	6 (8.11)
Histologic grade	
G1	12 (16.22)
G2	49 (66.22)
G3	13 (16.58)
Anatomical site	
Larynx	35 (47.30)
Oral cavity	39 (52.70)

### SASP factos transcript levels correlate with HNSCC patients’ clinical data

We analyzed the transcript level of selected SASP factors (*IL6*, *IL1b*, *CXCL1* and *TNF-α*) according to the patient’s clinical data: age at the time of surgery, gender, TNM classification, histologic grade, and anatomical site (Tables S2-S5).We found that *IL6* transcript level was significantly higher in cancerous tissues in both age groups (≤ 60 and > 60) and locations (larynx and oral cavity), but interestingly only in male patients and in T2,T3 and G2, G3, N0-N2 tumors (Table S2). Similarly, *IL1b* higher transcript level was revealed in both age groups (≤ 60 and > 60) and locations (larynx and oral cavity) in men but not women, in all histologic grade stages (G1-G3) and T3-T4 and N0, N2 TNM tumor stages (Table S3). *CXCL1* chemokine gene showed a difference in mRNA expression between the two examined tissues for almost every clinical feature (apart from T1, N3 and G1 tumors) (Table S4). In the case of *TNF-α* we observed significance difference in mRNA levels in men, patients over 60 years old with a tumor in both localizations, and in N0-N1, G1-G3 TNM stages (Table S5). We did not find a strong significant correlation between *p16* and *LMNB1* genes transcript level and patients' clinical data (Table S6-7).

### SASP factors transcript levels as potential clinical biomarkers

**Fig. 2:**
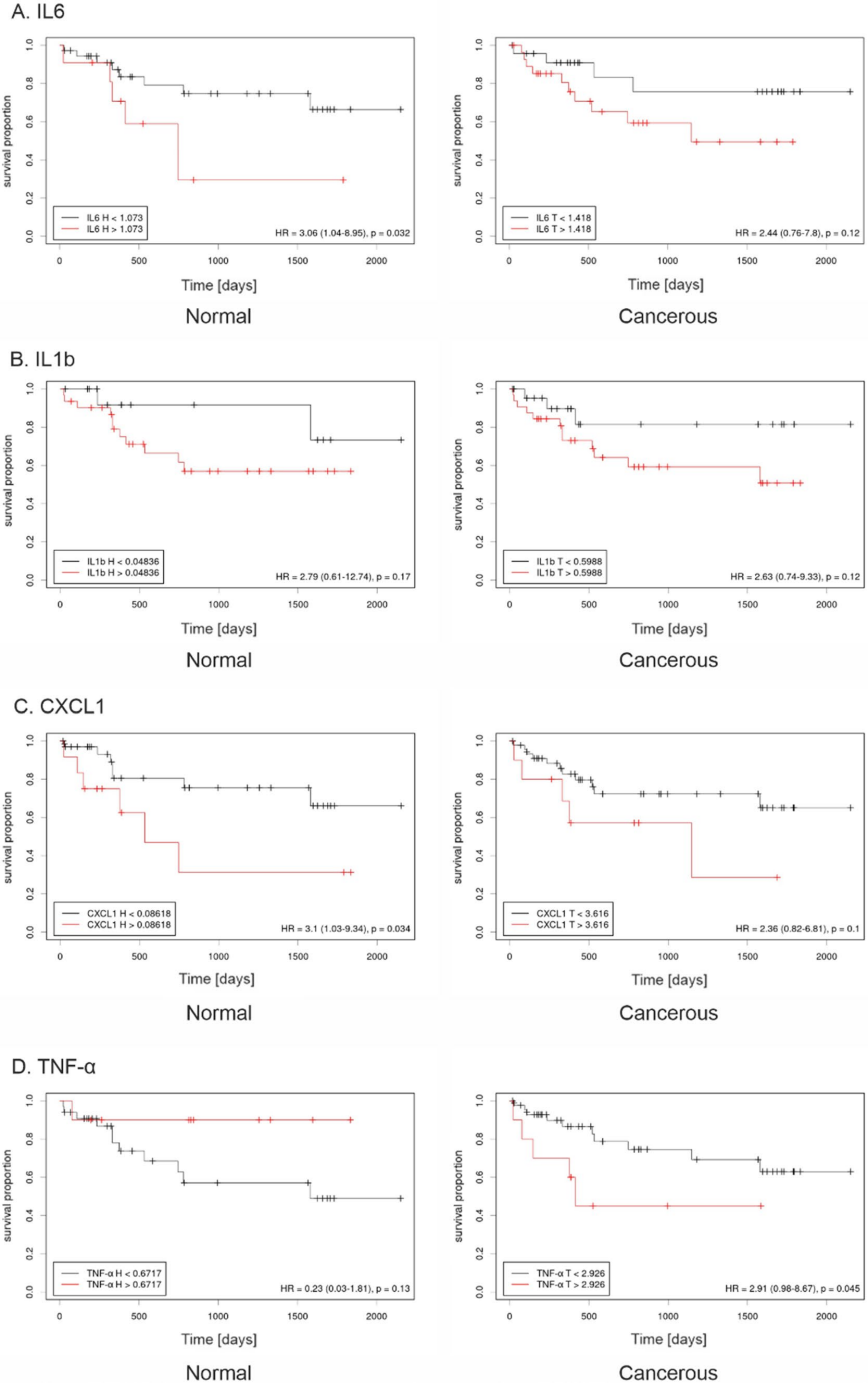
Transcript level of senescence-associated secretory phenotype (SASP) genes in cancerous and normal tissue correlates with the survival of HNSCC patients. The Kaplan–Meier survival analysis among patients with HNSCC was performed according to the expression of *IL6* (**A**); *IL1b* (**B**); *CXCL1* (**C**); TNF*-α* (**D**) in normal and cancerous tissue

To assess the effect of mRNA levels of selected SASP on patient outcomes we performed a retrospective analysis of 74 patients. The transcript levels of SASP (*IL6*, *IL1b*, *CXCL1* and *TNF-α*) and senescence markers (*p16, LMNB1*) were divided into two groups: low and high mRNA level determined by optimal cutoff points based on overall survival (OS). Kaplan–Meier analysis revealed a statistically significant increase of OS in patients with low level of *IL6* and *CXCL1* genes transcript in normal tissues, and low level of *TNF-α* transcript in cancerous tissues. In general, higher transcript levels of selected SASPs in cancerous tissues correlated with lower OS (Fig. 2). In turn, the higher transcript level of the *p16* senescent marker in normal tissues is significantly connected with higher OS (Figure S1).

For the analysis of SASP factors as biomarkers, we produced receiver operating characteristic (ROC) curves and considered the expression of SASP factors showing both sensitivity and specificity ≥ 0.8 as strong candidates, and SASPs showing both sensitivity and specificity ≥ 0.7 as potential, weaker candidates.

**Fig. 3:**
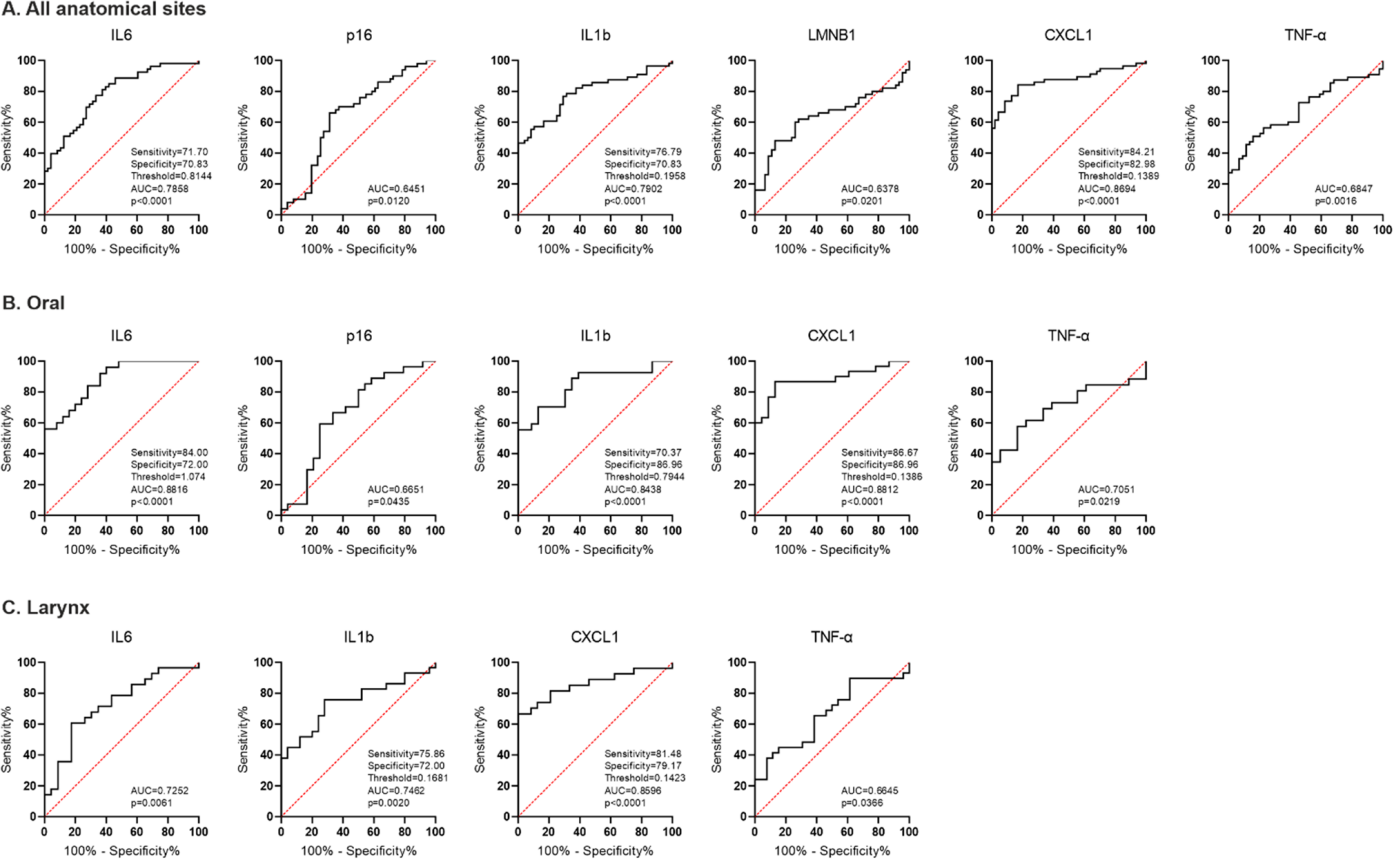
Transcript level of senescence-associated secretory phenotype (SASP) genes differentiates the normal and cancerous tissue of HNSCC patients. ROC curves were produced based on SASP biomarkers expression in all anatomical sites (**A**), oral (**B**), and laryngeal (**C**) cancer. AUC area under curve

Considering both tumor locations together (Fig. 3A), we have found *CXCL1* transcript level (sensitivity = 84.21, specificity = 82.98, ROC Area = 0.8694, threshold = 0.1389) as a strong candidate biomarker for distinguishing cancerous from normal tissue, and *IL6* (sensitivity = 71.70, specificity = 70.83, ROC Area = 0.7858, threshold = 0.8144) and *IL1b* (sensitivity = 76.79, specificity = 70.83, ROC Area = 0.7902, threshold = 0.1958) as weaker candidate biomarkers.

For oral cancer, *IL6* (sensitivity = 84, specificity = 72, ROC Area = 0.8816, threshold = 1.074) and *CXCL1* (sensitivity = 86.67, specificity = 86.96, ROC Area = 0.8812, threshold = 0.1386) expression reached the threshold sensitivity and specificity for a strong biomarker. While *IL1b* (sensitivity = 70.37, specificity = 86.96, ROC Area = 0.8438, threshold = 0.7944) expression reached the threshold sensitivity and specificity for weaker biomarker. (Fig. 3B). For laryngeal cancer, we found *CXCL1* (sensitivity = 81.48, specificity = 79.17, ROC Area = 0.8596, threshold = 0.1423) as a strong candidate and *IL1b* (sensitivity = 75.86, specificity = 72, ROC Area = 0.7462, threshold = 0.1681) as weak candidate biomarker. There were no statistically significant results of ROC curves for *p16* and *LMNB1* genes in all anatomical sites and for *TNF-α* in laryngeal location (Figure S2).

### CXCL1 SASP protein level in HNSCC patients’ serum

To investigate the role of CXCL1 chemokine as a non-invasive biomarker of HNSCC we analyzed the protein level in serum according to the patient’s clinical data: age at the time of surgery, gender, TNM classification, histologic grade, and anatomical site (Table 3). We found statistically significant higher CXCL1 serum levels in more histologically advanced (G2-G3) compared to G1 tumors. However, there was no correlation between transcript level of CXCL1 in tissue and its protein level in serum (Figure S3).

**Table 3:** CXCL1 protein levels in HNSCC patients’ serum (n = 40)

Characteristic	Serum, median (range)	p-value
Age at the time of surgery (years)		
≤ 60	4.935 (1.558–11.43)	0.6151^a^
> 60	2.792 (0.2597–11.95)	
Gender		
Male	2.792 (0.2597–11.95)	0.2916^a^
Female	5.974 (2.338–8.052)	
Tumor stage (TNM classification)		
T1	3.247 (1.558–4.935)	0.5116^b^
T2	2.987 (2.597–5.714)	
T3	2.208 (0.2597–11.95)	
T4	3.571 (1.429–11.43)	
N0	2.532 (1.169–11.95)	0.1093^b^
N1	4.286 (0.2597–11.30)	
N2	5.390 (2.597–11.43)	
N3	1.688 (1.429–1.948)	
Histologic grade		
G1	2.208 (1.169–4.935)	0.0119^b^
G2-G3	5.195 (2.597–7.013)	
Anatomical site		
Larynx	4.351 (1.818–11.95)	0.1564^a^
Oral cavity	2.792 (0.2597–11.43)	

The serum CXCL1 protein levels were measured in duplicates using ELISA. Depending on the data distribution we performed the U Mann–Whitney test^a^ or Kruskal–Wallis test^b^

## Discussion

A rising number of studies point out that spontaneous senescence and therapy-induced senescence (TIS) play a strong role in cancer aggressiveness. Senescent cells may have a role in oncogenesis mainly through the SASP, which produces an immunosuppressive environment. This aids in tumor development and relapse by secreting factors that contribute to cell proliferation, migration, invasiveness, angiogenesis, and epithelial-mesenchymal transition as well as immune-mediated clearance.

The main aim of this study was to quantify the selected SASP factors (*IL6, IL1b, CXCL1, TNF-α*) and senescence markers *(p16, LMNB1*) transcript expression and correlate this data with patients’ clinical features to clarify their physiological and pathological roles in HNSCC.

Our findings showed a significant increase in all selected SASP factors mRNA levels in cancerous tissue compared to normal tissue. The clinical features analyses indicate that higher transcript occurs mainly in men but in both age groups and localization as well as TNM stages and histologic grades. The difference between data for men and women might come from the fact that the men group was larger than women (60 vs. 14, respectively), and that males and females frequently differ in their rates of ageing.

The potential role of CXCL chemokines family in HNSCC was described in the study of Li et al., where they found a connection between different chemokines from the CXCL family and patient’s relapse free survival (RFS) or OS. Moreover, CXCL2, 3 and 12 were linked with low RFS; CXCL14 predicted increased RFS while CXCL9, 10, 14 and 17 showed higher OS, and CXCL1, 8 pointed towards a low OS [10]. Also, a meta-analysis in various cancers showed that a higher CXCL1 expression was positively correlated with a more advanced TNM stage and a higher likelihood of lymph node metastasis with a poor OS [11]. Moreover, we showed that CXCL1 chemokine proved to be a strong candidate as a tissue differentiating biomarker and tumor histological stage indicator from patients’ serum.

Interestingly, elevated IL-6 values in serum collected prior to nivolumab therapy of HNSCC at patients were correlated with their poor survival [12]. Also, Tsai et al. data revealed that IL-6 overexpression was associated with the increased risk of developing disease failure and poor prognosis for HNSCC [13]. Similarly, Jinno et al. elucidated the association of IL-6 expression with oral squamous cell carcinoma (OSCC) tumor progression, chemoresistance and prognosis [14]. These studies contribute to our findings regarding *IL-6* correlation to TNM stage, however there are differences concerning the transcript levels in normal versus cancerous tissue. We expect our results to drift towards a similar outcome with a more extensive study group.

While we did not see a higher *TNF-α* transcript level in laryngeal cancer patients and in all TNM stages, a study by Andersson et al. [15] pointed upregulation of *TNF-α* as potential biomarker of shorter patient survival independent of clinical stage.

IL1b is increased in a variety of malignancies, and it is well recognized that patients with overproduction of IL1b have poorer prognosis. Also, it has been demonstrated that IL1b is an indicator of cellular radioresistance and senescence in HNSCC cells without functional involvement in these processes [16]. In our study we saw a higher *IL1b* transcript level in both age groups (≤ 60 and > 60) and locations (larynx and oral cavity) in men but not women, in all histologic grade stages (G1-G3) and T3-T4 and N0, N2 TNM tumor stages. This data could potentially point toward a higher expression of *IL1b* in a higher TNM stage.

Our study did not show any correlation between head and neck cancer patients and *LMNB1* gene expression. However, it is positively upregulated in hepatocellular carcinoma correlating with the TNM stage, while in renal clear cell carcinoma high *LMNB1* levels exhibited poor prognosis [11].

In a study done by Jovanovic et al. on breast cancer increased expression of p16 indicated malignant transformation of non-invasive lesions and suggested that p16 can be used as an additional diagnostic test in separating benign from malignant changes [17]. In another study by Zhou et al. on colorectal cancer (CRC), *p16* overexpression was correlated with Dukes stage, lymph node metastasis, and TNM stage (only in Caucasians) suggesting its effect on the development of CRC [18]. In our study we did not see a statistically significant change of known senescent cell marker *p16* in normal versus cancerous tissue. We speculate that our observation of high SASPs expression in cancerous tissues may be induced not by p16 but by other senescent related pathways.

Given that many cancer treatments, especially radiation, can increase senescence and the SASP level [19], it is fair to infer that senescent cells may mediate a part of the short- and long-term deleterious effects of cancer treatments. Our study shows new and potential biomarkers that have previously not been used, even though the idea of molecular biomarkers in HNSCC has been around for some time. Since the discovery of senescent cells, researchers have had difficulty identifying common and clear-cut markers that define the senescence state. The complexity of the senescence phenotype and the existence of extremely diverse senescence programs are reflected in the challenge of discovering such markers [20–22]. Yet, these studies are of high significance to many present cancerous and non-cancerous diseases associated with cellular senescence, especially in the current development of senescent targeted therapies using novel “senolytic” drugs [22]. These groups of drugs can selectively target and stimulate the apoptosis of senescent cells. Beside large numbers of studies in different animal models, there are ongoing clinical trials already indicate patient safety, health improvement, and success in clearing senescent cells from different tissues [23–25].

The limitation of our study is a quite small study group and a lack of description of the molecular mechanisms and consequences of SASPs expression for tumorigenesis in HNSCC in vitro or in vivo models. Moreover, a more detailed study is needed to present specific role of senescence-related mechanism and SASPs in tumor therapy response in relation to the condition of the patient’s immune system. Furthermore, due to patients’ material limitations, we have not determined the protein level of the selected SASP factors. Nevertheless, further large-scale, longitudinal studies are needed to confirm the clinical relevance of our observation. Moreover, the high transcript level of selected SASP factors (*IL6, IL1b, CXCL1, TNF-α*) in cancerous tissues suggests their role in HNSCC development which could point future targeting of senescent cells as a secondary treatment might be beneficial for HNSCC patients. Furthermore, our findings indicate CXCL1 chemokine as a strong candidate as a tissue differentiating biomarker and tumor histological stage indicator from patients’ serum, and IL1b overexpression as a molecular factor related to increased TNM stage.

## Conclusions

From the above discussion, the SASP factors as potential diagnostic biomarkers may play a key role in understanding their effects on tumor recurrence, patient survival, and patient response to treatment. The elevated levels of *CXCL1*, *IL-6*, *IL1b*, and *TNF-α* in cancerous tissue compared to normal tissue and its correlation with clinicopathological features and survival of HNSCC patients show a promising approach to identifying biomarkers for the advancement of the disease and differentiation between the two types of tissues. As a result, the SASP harmful, chronic inflammatory effects may take time to manifest. It is thus critical to understand the mechanisms that govern the SASP to know how it might be adjusted and whether the potentially harmful effects of the SASP can be reduced without jeopardizing the beneficial effects of senescence. The main limitation of our study is lack of explanation no scientific evidence why expression varies with different clinical features. Also, we proposed CXCL1 and IL1b as the most relevant factors examined, but we did not test their deficiency and excess in cellular models. Further studies with a larger sample size are needed to understand this process better and the mechanistic role of senescent cells, which will expedite the inclusion of HNSCC individuals in future clinical trials with newly developed senolytics.

### Supplementary Information

Below is the link to the electronic supplementary material.

Supplementary file1 (DOCX 433 KB)

## Data Availability

The datasets generated and analysed during the current study are not publicly available due to the sensitive nature of the data but are available from the corresponding author on reasonable request.

## Abbreviations

CRC: Colorectal cancer
CXCL1: C-X-C motif chemokine ligand 1
CCL: C–C motif chemokine ligand
EMT: Epithelial–mesenchymal transition
FDA: Food and drug administrartion
HNSCC: Head and neck squamous cell carcinoma
IL1b: Interleukin 1b
IL6: Interleukin 6
LMNB1: Lamin B1
OS: Overall survival
OSCC: Oral squamous cell carcinoma
p16: Cyclin-dependent kinase inhibitor 2A
RFS: Relapse free survival
RT-qPCR: Real-time quantitative polymerase chain reaction
SASP: Senescence-associated secretory phenotype
SMS: Senescence-messaging secretome
TIS: Therapy-induced Senescence
TME: Tumor microenvironment
TNF-α: Tumor necrosis factor α
