# Supplementary material for: Senescence in head and neck squamous cell carcinoma: relationship between senescence-associated secretory phenotype (SASP) mRNA expression level and clinicopathological features
Source: Clin Transl Oncol. 2024 Jan 4;26(4):1022–32. doi: 10.1007/s12094-023-03364-6 (PMC10981631; doi:10.1007/s12094-023-03364-6)
Supplement: Supplementary file 1 — Supplementary file1 (DOCX 433 KB) [file 12094_2023_3364_MOESM1_ESM.docx]

**Supplementary material**

**Table S1.** Oligonucleotide sequences used in qPCR analysis

| **Gene** | **Sequence (5’-3’)** |
| --- | --- |
| 18S rRNA | F: CTACCACATCCAAGGAAGCA  R: TTTTTCGTCACTACCTCCCCG |
| IL6 | F: ACTCACCTCTTCAGAACGAATTG  R: CCATCTTTGGAAGGTTCAGGTTG |
| IL1b | F: CTCGCCAGTGAAATGATGGCT  R: GTCGGAGATTCGTAGCTGGAT |
| CXCL1 | F: GCGCCCAAACCGAAGTCATA  R: ATGGGGGATGCAGGATTGAG |
| TNF-α | F: CCTCTCTCTAATCAGCCCTCTG  R: GAGGACCTGGGAGTAGATGAG |
| LMNB1 | F: TTGGATGCTCTTGGGGTTC  R: AAGCAGCTGGAGTGGTTGTT |
| P16 | F: CCAACGCACCGAATAGTTACG  R: GCGCTGCCCATCATCATG |

**Table S2.** The comparison of *IL6* transcript levels in cancerous (n=72) and normal (n=64) tissues from HNSCC patients grouped according to clinical features.

| **Characteristic** | **Normal tissue** | **Cancerous tissue** | **p-value** |
| --- | --- | --- | --- |
|  | **Median (range) ^a^ or** | **Median (range) ^a^ or** |  |
|  | **Mean (± SD) ^b^** | **Mean (± SD) ^b^** |  |
| **Age at the time of surgery (years)** | | | |
| ≤60 | 0.15 (0.02-3.10)^a^ | 1.47 (0.01-10.52)^a^ | 0.0027 |
| >60 | 0.26 (0.01-4.21)^a^ | 1.98 (0.07-20.73)^a^ | <0.0001 |
| **Gender** | | | |
| Male | 0.24 (0.01-4.21)^a^ | 1.97 (0.07-16.41)^a^ | <0.0001 |
| Female | 0.13 (0.05-4.07)^a^ | 1.47 (0.01-20.73)^a^ | 0.1011 |
| **Tumor stage (TNM classification)** | | | |
| T1 | 0.05 (0.02-3.1)^a^ | 1.97 (0.78-8.07)^a^ | 0.2 |
| T2 | 0.62 (±0.61)^b^ | 4.88 (±4.74)^b^ | 0.0238 |
| T3 | 0.21 (0.01-3.07)^a^ | 1.22 (0.09-20.73)^a^ | 0.0009 |
| T4 | 0.3 (0.05-4.21)^a^ | 1.15 (0.01-9.74)^a^ | 0.0574 |
| N0 | 0.22 (0.01-3.1)^a^ | 0.89 (0.09-8.07)^a^ | 0.0025 |
| N1 | 0.21 (0.02-2.16)^a^ | 1.33 (0.12-16.41)^a^ | 0.0011 |
| N2 | 0.09 (0.02-4.21)^a^ | 1.76 (0.01-20.73)^a^ | 0.0161 |
| N3 | 1.41 (±0.36)^b^ | 6.68 (±5.78)^b^ | 0.1187 |
| **Histologic grade** | | | |
| G1 | 0.21 (0.01-3.07)^a^ | 0.91 (0.12-16.41)^a^ | 0.0907 |
| G2 | 0.24 (0.02-4.07)^a^ | 1.47 (0.01-20.73)^a^ | <0.0001 |
| G3 | 0.21 (0.05-4.21)^a^ | 2.04 (0.17-8.4)^a^ | 0.0188 |
| **Anatomical site** | | | |
| Larynx | 0.21 (0.04-4.21)^a^ | 0.82 (0.01-8.4)^a^ | 0.0055 |
| Oral cavity | 0.25 (0.01-3.1)^a^ | 3.45 (0.57-20.73)^a^ | <0.0001 |

The *IL6* transcript levels were measured in triplicates and standardized by *18S* rRNA reference gene, relative gene expression was calculated using the Pfaffl method. Depending on the data distribution we performed the U Mann-Whitney test^a^ or unpaired t-test^b^.

**Table S3.** The comparison of *IL1b* transcript levels in cancerous (n=72) and normal (n=64) tissues from HNSCC patients grouped according to clinical features.

| **Characteristic** | **Normal tissue** | **Cancerous tissue** | **p-value** |
| --- | --- | --- | --- |
|  | **Median (range) ^a^ or** | **Median (range) ^a^ or** |  |
|  | **Mean (± SD) ^b^** | **Mean (± SD) ^b^** |  |
| **Age at the time of surgery (years)** | | | |
| ≤60 | 0.08 (0.008-0.76)^a^ | 0.68 (0.003-7.44)^a^ | 0.0101 |
| >60 | 0.14 (0.009-1.24)^a^ | 1.15 (0.008-11.69)^a^ | <0.0001 |
| **Gender** | | | |
| Male | 0.12 (0.008-1.24)^a^ | 0.87 (0.003-11.69)^a^ | <0.0001 |
| Female | 0.3 (0.009-1.24)^a^ | 0.77 (0.03-8.84)^a^ | 0.1229 |
| **Tumor stage (TNM classification)** | | | |
| T1 | 0.01 (0.01-0.12)^a^ | 0.95 (0.15-7.37)^a^ | 0.1 |
| T2 | 0.33 (0.038-0.76)^a^ | 1.48 (0.16-3.97)^a^ | 0.2284 |
| T3 | 0.12 (0.009-1.1)^a^ | 1.15 (0.003-11.69)^a^ | 0.0038 |
| T4 | 0.12 (0.008-1.24)^a^ | 0.49 (0.008-7.44)^a^ | 0.0025 |
| N0 | 0.04 (0.008-1.24)^a^ | 0.6 (0.01-8.84)^a^ | 0.0001 |
| N1 | 0.16 (0.1-0.96)^a^ | 1.46 (0.003-11.69)^a^ | 0.0831 |
| N2 | 0.1 (0.009-1.24)^a^ | 1.15 (0.008-4.23)^a^ | 0.0119 |
| N3 | 0.29 (±0.36)^b^ | 1.35 (±1.35)^b^ | 0.3 |
| **Histologic grade** | | | |
| G1 | 0.05 (0.01-0.53)^a^ | 1.66 (0.04-8.84)^a^ | 0.0164 |
| G2 | 0.13 (0.008-1.24)^a^ | 0.91 (0.003-11.69)^a^ | 0.0004 |
| G3 | 0.14 (0.04-0.71)^a^ | 0.55 (0.09-4.23)^a^ | 0.0057 |
| **Anatomical site** | | | |
| Larynx | 0.14 (0.008-0.96)^a^ | 0.43 (0.003-8.84)^a^ | 0.0027 |
| Oral cavity | 0.12 (0.01-1.24)^a^ | 1.28 (0.01-11.69)^a^ | <0.0001 |

The *IL1b* transcript levels were measured in triplicates and standardized by *18S* rRNA reference gene, relative gene expression was calculated using the Pfaffl method. Depending on the data distribution we performed the U Mann-Whitney test^a^ or unpaired t-test^b^.

**Table S4.** The comparison of *CXCL1* transcript levels in cancerous (n=72) and normal (n=64) tissues from HNSCC patients grouped according to clinical features.

| **Characteristic** | **Normal tissue** | **Cancerous tissue** | **p-value** |
| --- | --- | --- | --- |
|  | **Median (range) ^a^ or** | **Median (range) ^a^ or** |  |
|  | **Mean (± SD) ^b^** | **Mean (± SD) ^b^** |  |
| **Age at the time of surgery (years)** | | | |
| ≤60 | 0.02 (0.005-0.25)^a^ | 1.19 (0.001-6.21)^a^ | 0.0024 |
| >60 | 0.03 (0.002-0.4)^a^ | 0.94 (0.02-8.74)^a^ | <0.0001 |
| **Gender** | | | |
| Male | 0.02 (0.002-0.4)^a^ | 1.25 (0.001-8.74)^a^ | <0.0001 |
| Female | 0.03 (0.003-0.36)^a^ | 0.4 (0.01-4.08)^a^ | 0.0031 |
| **Tumor stage (TNM classification)** | | | |
| T1 | 0.02 (0.01-0.019)^a^ | 1.41 (0.01-3.02)^a^ | 0.2667 |
| T2 | 0.02 (0.01-0.4)^a^ | 2.82 (0.16-6.21)^a^ | 0.0012 |
| T3 | 0.01 (0.002-0.36)^a^ | 1.23 (0.01-7.39)^a^ | <0.0001 |
| T4 | 0.03 (0.005-0.36)^a^ | 0.53 (0.001-8.74)^a^ | 0.0001 |
| N0 | 0.02 (0.002-0.36)^a^ | 1.18 (0.001-8.74)^a^ | <0.0001 |
| N1 | 0.04 (0.002-0.36)^a^ | 0.37 (0.003-7.39)^a^ | <0.0001 |
| N2 | 0.02 (0.003-0.18)^a^ | 1.63 (0.14-6.23)^a^ | <0.0001 |
| N3 | 0.18 (±0.2)^b^ | 1.41 (±1.15)^b^ | 0.1335 |
| **Histologic grade** | | | |
| G1 | 0.01 (0.002-0.36)^a^ | 0.31 (0.01-5.39)^a^ | 0.0513 |
| G2 | 0.02 (0.002-0.4)^a^ | 0.77 (0.001-6.23)^a^ | <0.0001 |
| G3 | 0.06 (0.007-0.18)^a^ | 1.66 (0.14-8.74)^a^ | <0.0001 |
| **Anatomical site** | | | |
| Larynx | 0.03 (0.005-0.36)^a^ | 0.57 (0.001-8.74)^a^ | <0.0001 |
| Oral cavity | 0.02 (0.002-0.4)^a^ | 1.08 (0.003-6.21)^a^ | <0.0001 |

The *CXCL1* transcript levels were measured in triplicates and standardized by *18S* rRNA reference gene, relative gene expression was calculated using the Pfaffl method. Depending on the data distribution we performed the U Mann-Whitney test^a^ or unpaired t-test^b^.

**Table S5.** The comparison of *TNF-α* transcript levels in cancerous (n=72) and normal (n=64) tissues from HNSCC patients grouped according to clinical features.

| **Characteristic** | **Normal tissue** | **Cancerous tissue** | **p-value** |
| --- | --- | --- | --- |
|  | **Median (range) ^a^ or** | **Median (range) ^a^ or** |  |
|  | **Mean (± SD) ^b^** | **Mean (± SD) ^b^** |  |
| **Age at the time of surgery (years)** | | | |
| ≤60 | 0.21 (0.03-1.89)^a^ | 0.51 (0.009-6.15)^a^ | 0.0832 |
| >60 | 0.38 (0.01-2.02)^a^ | 1.15 (0.0005-6.64)^a^ | 0.006 |
| **Gender** | | | |
| Male | 0.23 (0.005-2.02)^a^ | 0.81 (0.0005-6.64)^a^ | 0.0023 |
| Female | 0.57 (0.12-1.63)^a^ | 1.01 (0.04-6.15)^a^ | 0.4278 |
| **Tumor stage (TNM classification)** | | | |
| T1 | 0.42 (0.05-0.78)^a^ | 0.62 (0.25-0.99)^a^ | 0.6667 |
| T2 | 0.22 (0.1-1.89)^a^ | 1.19 (0.35-4.36)^a^ | 0.0553 |
| T3 | 0.4 (0.005-1.63)^a^ | 0.77 (0.003-6.15)^a^ | 0.2968 |
| T4 | 0.23 (0.02-2.02)^a^ | 0.37 (0.0005-6.64)^a^ | 0.0417 |
| N0 | 0.11 (0.01-1.89)^a^ | 0.57 (0.004-4.41)^a^ | 0.0071 |
| N1 | 0.39 (0.06-0.78)^a^ | 0.65 (0.003-4.06)^a^ | <0.0001 |
| N2 | 0.57 (0.06-2.02)^a^ | 1.15 (0.0005-6.34)^a^ | 0.1675 |
| N3 | 0.26 (0.25-0.26)^a^ | 4.36 (0.3-6.64)^a^ | 0.0952 |
| **Histologic grade** | | | |
| G1 | 0.71 (±0.5)^b^ | 0.96 (±1.05)^b^ | 0.5859 |
| G2 | 0.2 (0.005-2.02)^a^ | 0.66 (0.0005-6.64)^a^ | 0.0083 |
| G3 | 0.4 (±0.22)^b^ | 2.13 (±1.99)^b^ | 0.026 |
| **Anatomical site** | | | |
| Larynx | 0.22 (0.005-2.02)^a^ | 0.34 (0.0005-6.34)^a^ | 0.0366 |
| Oral cavity | 0.42 (0.05-1.89)^a^ | 1.16 (0.003-6.64)^a^ | 0.0215 |

The *TNF-α* transcript levels were measured in triplicates and standardized by *18S* rRNA reference gene, relative gene expression was calculated using the Pfaffl method. Depending on the data distribution we performed the U Mann-Whitney test^a^ or unpaired t-test^b^.

**Table S6.** The comparison of *P16* transcript levels in cancerous (n=72) and normal (n=64) tissues from HNSCC patients grouped according to clinical features.

| **Characteristic** | **Normal tissue** | **Cancerous tissue** | **p-value** |
| --- | --- | --- | --- |
|  | **Median (range) ^a^ or** | **Median (range) ^a^ or** |  |
|  | **Mean (± SD) ^b^** | **Mean (± SD) ^b^** |  |
| **Age at the time of surgery (years)** | | | |
| ≤60 | 0.17 (0.001-0.96)^a^ | 0.02 (0.0002-2.12)^a^ | 0.2711 |
| >60 | 0.29 (0.002-1.81)^a^ | 0.11 (0.004-2.18)^a^ | 0.1667 |
| **Gender** | | | |
| Male | 0.24 (0.002-1.81)^a^ | 0.07 (0.0008-2.17)^a^ | 0.0636 |
| Female | 0.17 (0.001-1.74)^a^ | 0.23 (0.0002-2.18)^a^ | 0.882 |
| **Tumor stage (TNM classification)** | | | |
| T1 | 0.004 (0.001-0.39)^a^ | 0.008 (0.0002-0.11)^a^ | >0.9999 |
| T2 | 0.25 (0.02-1.74)^a^ | 0.23 (0.02-2.12)^a^ | 0.7972 |
| T3 | 0.28 (0.002-1.81)^a^ | 0.02 (0.002-2.17)^a^ | 0.0158 |
| T4 | 0.2 (0.002-1.25)^a^ | 0.16 (0.0008-2.18)^a^ | 0.8701 |
| N0 | 0.09 (0.001-1.81)^a^ | 0.03 (0.0002-2.18)^a^ | 0.3983 |
| N1 | 0.29 (0.008-1.74)^a^ | 0.07 (0.007-2.17)^a^ | 0.4923 |
| N2 | 0.32 (0.004-1.25)^a^ | 0.17 (0.002-2.12)^a^ | 0.5179 |
| N3 | 0.48 (0.13-0.84)^a^ | 0.07 (0.02-0.9)^a^ | 0.4286 |
| **Histologic grade** | | | |
| G1 | 0.35 (0.001-1.81)^a^ | 0.3 (0.0002-0.33)^a^ | 0.0279 |
| G2 | 0.25 (0.002-1.74)^a^ | 0.08 (0.0008-2.18)^a^ | 0.6403 |
| G3 | 0.19 (±0.11)^b^ | 0.18 (±0.14)^b^ | 0.8354 |
| **Anatomical site** | | | |
| Larynx | 0.23 (0.002-1.62)^a^ | 0.09 (0.0008-2.06)^a^ | 0.2046 |
| Oral cavity | 0.22 (0.001-1.81)^a^ | 0.06 (0.0002-2.18)^a^ | 0.2217 |

The *p16* transcript levels were measured in triplicates and standarized by 18S rRNA reference gene, relative gene expression were calculate using Pfaffl method. Depending on the data distribution we performed the U Mann-Whitney test^a^ or unpaired t-test^b^.

**Table S7.** *LMNB1* transcript levels in in cancerous (n=72) and normal (n=64) tissues samples from HNSCC patients.

| **Characteristic** | **Normal tissue** | **Cancerous tissue** | **p-value** |
| --- | --- | --- | --- |
|  | **Median (range) ^a^ or** | **Median (range) ^a^ or** |  |
|  | **Mean (± SD) ^b^** | **Mean (± SD) ^b^** |  |
| **Age at the time of surgery (years)** | | | |
| ≤60 | 0.16 (0.04-0.93)^a^ | 0.53 (0.002-2.46)^a^ | 0.3392 |
| >60 | 0.31 (0.02-0.89)^a^ | 0.41 (0.003-2.22)^a^ | 0.0583 |
| **Gender** | | | |
| Male | 0.24 (0.02-0.89)^a^ | 0.43 (0.002-2.22)^a^ | 0.0086 |
| Female | 0.44 (0.04-0.93)^a^ | 0.42 (0.05-2.46)^a^ | 0.743 |
| **Tumor stage (TNM classification)** | | | |
| T1 | 0.14 (0.09-0.18)^a^ | 0.16 (0.1-0.8)^a^ | 0.8 |
| T2 | 0.22 (±0.16)^b^ | 0.74 (±0.56)^b^ | 0.0539 |
| T3 | 0.24 (0.04-0.89)^a^ | 0.61 (0.02-2.46)^a^ | 0.0544 |
| T4 | 0.26 (0.07-0.93)^a^ | 0.41 (0.002-2.22)^a^ | 0.3951 |
| N0 | 0.15 (0.02-0.89)^a^ | 0.41 (0.003-1.92)^a^ | 0.0611 |
| N1 | 0.22 (0.08-0.48)^a^ | 0.29 (0.002-2.22)^a^ | 0.3148 |
| N2 | 0.34 (0.09-0.93)^a^ | 0.51 (0.02-2.46)^a^ | 0.3394 |
| N3 | 0.18 (0.04-0.32)^a^ | 1.05 (0.68-1.43)^a^ | 0.3333 |
| **Histologic grade** | | | |
| G1 | 0.31 (±0.13)^b^ | 0.67 (±0.74)^b^ | 0.2729 |
| G2 | 0.24 (0.07-0.93)^a^ | 0.52 (0.002-2.22)^a^ | 0.0591 |
| G3 | 0.23 (0.02-0.88)^a^ | 0.41 (0.003-2.46)^a^ | 0.1564 |
| **Anatomical site** | | | |
| Larynx | 0.28 (0.07-0.93)^a^ | 0.42 (0.003-2.46)^a^ | 0.1756 |
| Oral cavity | 0.23 (0.02-0.58)^a^ | 0.49 (0.002-1.92)^a^ | 0.08 |

The *LMNB1* transcript levels were measured in triplicates and standarized by 18S rRNA reference gene, relative gene expression were calculate using Pfaffl method. Depending on the data distribution we performed the U Mann-Whitney test^a^ or unpaired t-test^b^.

**
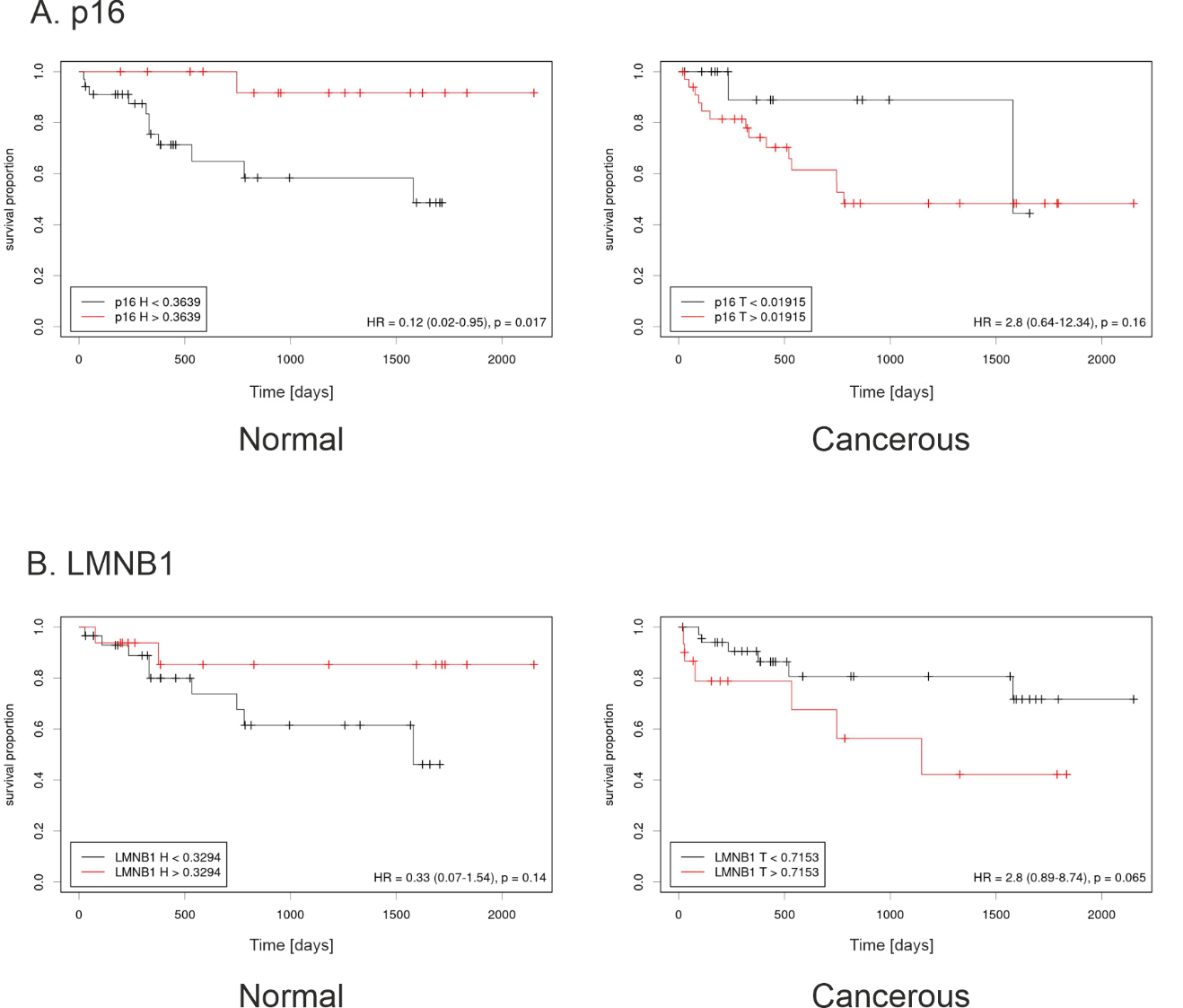
**

**Figure S1. The Kaplan–Meier survival analysis among patients with HNSCC according to the expression of senescence marker genes:** P16(A); LMNB1(B).

**
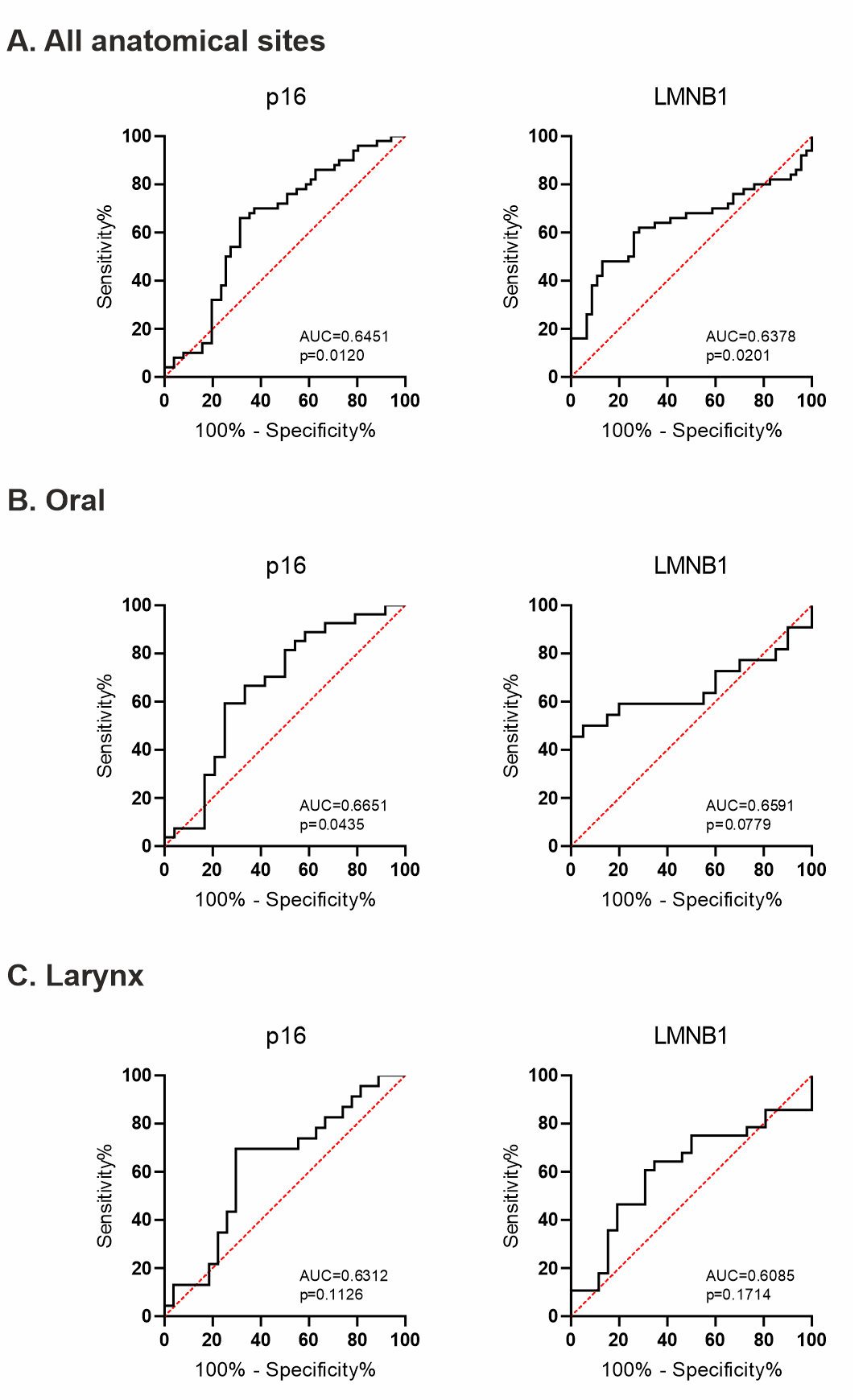
**

**Figure S2.** ROC curves differentiating between normal and cancerous tissue for LMNB1 in oral (A) and of p16 and LMNB1 in laryngeal (B) tissues.

**
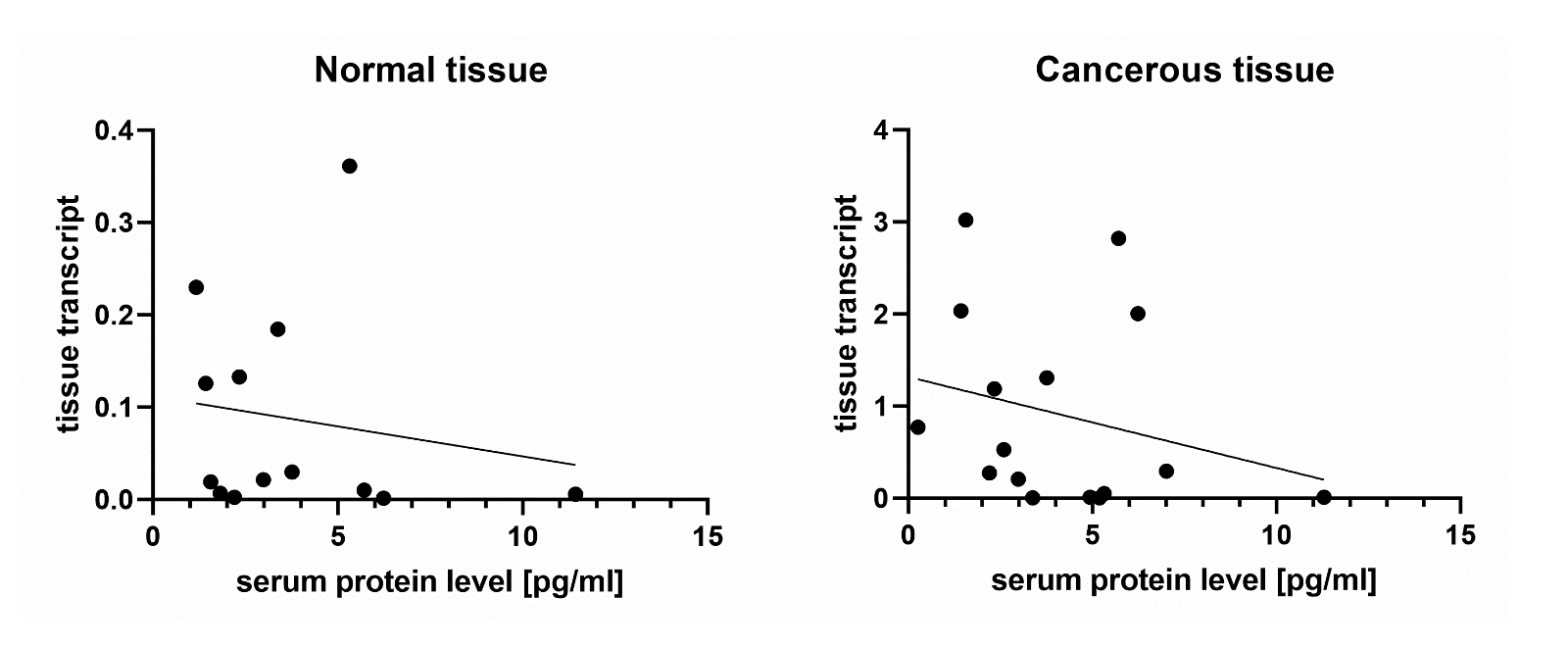
**

**Figure S3.** Spearman’s correlation between CXCL1 tissue transcript level (in normal and cancerous tissue from HNSCC patients) and CXCL1 serum protein level.
